# Supplementary material for: Failure to receive prescribed imaging is associated with increased early mortality after injury in Cameroon
Source: PLOS Glob Public Health. 2023 Aug 18;3(8):e0001951. doi: 10.1371/journal.pgph.0001951 (PMC10437996; doi:10.1371/journal.pgph.0001951)
Supplement: Attachment — Submitted filename: Response to Reviewers.pdf [file pgph.0001951.s002.pdf]

UNIVERSITY OF CALIFORNIA, LOS ANGELES **UCLA**

BERKELEY • DAVIS • IRVINE • LOS ANGELES • MERCED • RIVERSIDE • SAN DIEGO • SAN FRANCISCO SANTA BARBARA • SANTA CRUZ

DEPARTMENT OF SURGERY
DAVID GEFFEN SCHOOL OF MEDICINE, UCLA
10833 LE CONTE AVE 72-215 CHS
LOS ANGELES, CALIFORNIA 90095-6904
PHONE: (310) 267-9607
FAX: (310) 267-3590

April 28, 2023

Dear Dr. Mowafi,

Thank you for your review of our manuscript "Failure to Receive Prescribed Imaging Portends Increased Early Mortality after Injury in Cameroon" for PLOS Global Public Health. We appreciate the insightful comments presented by the reviewers and appreciate this opportunity to strengthen our submission. Below, we have responded to each comment and have attached a revised manuscript incorporating our revisions (both highlighted tracked changes and an unmarked version).
We appreciate your time and ongoing consideration of our manuscript for publication in PLOS Global Public Health. Please do not hesitate to contact us for any further questions or comments.

Sincerely,

Matthew Driban, BS (Corresponding Author)

Please see below for an itemized response to each of the reviewers' comments.

Journal Requirements:

1. **In the ethics statement in the Methods, you have specified that verbal consent was obtained. Please provide additional details regarding how this consent was documented and witnessed, and state whether this was approved by the IRB**

Thank you. A standard oral consent script is administered to all injured patients or their surrogates by Cameroonian national research assistants. Patients/surrogates are given the opportunity to ask questions and, if obtained, consent is documented. Verbal consent is obtained due to variable rates of patient literacy. To increase clarity of our manuscript we have provided additional protocol details regarding the informed consent process of the Cameroon Trauma Registry. Furthermore, we have documented IRB approval (Methods, lines 100-109).

2. **Please send a completed 'Competing Interests' statement, including any COIs declared by your co-authors. If you have no competing interests to declare, please state "The authors have declared that no competing interests exist". Otherwise please declare all competing interests beginning with the statement "I have read the journal's policy and the authors of this manuscript have the following competing interests:"**

Thank you, we have added a 'Competing Interests' statement (Line 315).

3. **Please amend your detailed Financial Disclosure statement. This is published with the article. It must therefore be completed in full sentences and contain the exact wording you wish to be published.**
    a. **State the initials, alongside each funding source, of each author to receive each grant.**
    b. **State what role the funders took in the study. If the funders had no role in your study, please state: "The funders had no role in study design, data collection and analysis, decision to publish, or preparation of the manuscript."**
    c. **If any authors received a salary from any of your funders, please state which authors and which funders. If you did not receive any funding for this study, please simply state: "The authors received no specific funding for this work."**

Thank you, we have modified our Financial Disclosure statement to include the requested details. We have also addended the statement to the end of our revised manuscript (Lines 316-320).

4. **We do not publish any copyright or trademark symbols that usually accompany proprietary names, eg ©, ®, ™ (e.g. next to drug or reagent names). Please remove all instances of trademark/copyright symbols throughout the text, including ® on page 15.**

Thank you for the clarification. We have removed the trademark symbol from our manuscript (References, item 9, lines 349-351).

5. **In the online submission form, you indicated that "Data available upon request from the authors". All PLOS journals now require all data underlying the findings described in their manuscript to be freely available to other researchers, either 1. In a public repository, 2. Within the manuscript itself, or 3. Uploaded as supplementary information. This policy applies to all data except where public deposition would breach compliance with the protocol approved by your research ethics board. If your data cannot be made publicly available for ethical or legal reasons (e.g., public availability would compromise patient privacy), please explain your reasons by return email and your exemption request will be escalated to the editor for approval. Your exemption request will be handled independently and will not hold up the peer review process, but will need to be resolved should your manuscript be accepted for publication. One of the Editorial team will then be in touch if there are any issues.**

Thank you. We have uploaded our de-identified dataset entitled "CTR_Radiology_PLOS_Deidentified.xslx" as a supplementary file.

Reviewer 1:

1. **"70 reportedly at least one government-funded imaging center in each of Cameroon's 10 geopolitical 71 regions offers computed tomography, imaging capacity is largely concentrated in three major 72 urban areas: Yaoundé, Douala, and Bafoussam. Over 80% of Cameroonian imaging centers are 73 limited to radiographs and/or ultrasound. Most existing equipment is donated, refurbished, or has 74 been in use for over 10 years." The above statement requires a citation (Lines 70-74).**

We would like to thank the reviewers for their comment. We have added the citation to the manuscript (Reference section, item 15, line 76).

2. **Ethics Statement "Verbal consent was obtained by research assistant" (Line 97) This raises some concern of data safety. What is the procedure for obtaining consent for use of the registry? It is unlikely that it would be standard procedure to obtain verbal permission from Cameroon NEC, and UCLA Institutional Review Boards for use of this data. Authors should kindly review this, and provide an exemption IRB certificate or documented Ethics Approval at the least. If no formal permission has been obtained, the authors should consider a retrospective approval so as to confirm that local partners are agreed to the use of this data. This is a major concern affecting the reviewer decision.**

A standard oral consent script is administered to all injured patients or their surrogates by Cameroonian national research assistants. Patients/surrogates are given the opportunity to ask questions and, if obtained, consent is documented. Verbal consent is obtained to maximize inclusion due to variable rates of literacy among Cameroonian trauma patients. To increase clarity of our manuscript we have provided additional protocol details regarding the informed consent process of the Cameroon Trauma Registry. Furthermore, we have documented IRB approval (Methods, lines 100-109).

3. **Lines 111-112- "access to liquid petroleum gas (LPG) was used as a surrogate indicator of higher socioeconomic status" The limitations of using this should be highlighted in limitations as more accurate measures of SES could have been collected.**

We would like to thank the reviewers for their comment. We completely agree with the reviewer that LPG utilization is only an indicator, and certainly not a comprehensive evaluation, of socioeconomic status in our study population. Our group has previously published methodology for using LPG utilization and four other demographic and health survey indicator questions to generate socioeconomic clusters that correlate tightly with socioeconomic outcome metrics in Cameroon. To increase clarity, we have emphasized that LPG use is only an indicator and provided greater detail and a citation to the methods (line 125) and limitations sections (lines 304-306.

4. **Line 135 Table 1. Demographics of Injured Patients Authors suggest missing demographics data in limitations but no recourse is made to how this was handled. Are these percentages of the total (n)- in which case, no demographics data is missing- or percentages of varying (n)s based on data is available for each demographic consideration? Authors to please clarify in the text or in the case of handling of missingness, in the table.**

We would like to thank the reviewers for their comment. In cases of data missingness, we used variable n values and clarified this recourse in the limitations section (Limitations, lines 295-297).

5. **Line 136 IQR, interquartile range. IQR is not referenced in table 1 and should not be in the footnotes**

We would like to thank the reviewers for their comment. We reference IQR with median age in Table 1.

6. **173 to 174- The authors noted that "NC patients reported significantly higher rate of cost interference with care (60.5%) compared to COMPLETED" Could this be because patients could afford the investigations? A higher capacity to pay results in better care and not just in receipt of more imaging which implies a likelihood to progress in care. As NC patients reported a "significantly higher rate of cost interference with care (60.5%) compared to COMPLETED patients" Line 181- "NC patients left against medical advice (35.0% vs. 21.3%, p<0.01) or died**

(5.6% vs. 1.3%, 182 p<0.01) more frequently" Authors should look into the sequence- did NC have less imaging because they died before imaging was possible? or left against medical advice before imaging was available? Or physicians considered it medical futility to carry out some investigations in these patients? Is there a threshold of days on admission considered, as a subset of the analysis to ensure that early deaths do not confound these results? Can this be emphasized? Kindly add in a time to death (in NC) versus time to intervention in C to clarify.

We would like to thank the reviewers for their questions. We absolutely agree that all of these suggestions are possibilities. Unfortunately, a notable limitation of the observational methodology employed in this study is that it is not possible to infer causality between these associated events. We hypothesize that inability to pay is indeed a significant contributing factor in both failure to complete prescribed imaging and in failure to receive other types of care, all of which likely contribute to poor outcomes including increased rates of death. To support scaling efforts, we think it is critical to further explore the impact on patient outcomes when diagnostic imaging is available by performing a controlled clinical trial. We also hypothesize that there is likely some survivorship bias to completing prescribed imaging among those who do not die or leave against medical advice. However, we still feel the association highlights a critical and possibly actionable care delivery deficit in the Cameroon Trauma System as in developed trauma settings, diagnostic imaging (radiographs and point-of care ultrasonography) is performed as a part of the initial trauma survey and assessment, within the first minutes of the patient presenting for evaluation. Average time to death in the whole study cohort was 3.9 hours, which in an optimized setting would provide ample time for completion of at least some imaging. We have added data on time to intervention and death in results (Results, lines 201-205) and emphasized these points in our discussion (Discussion, lines 248-252).

7. **Line 185 "Time to disposition did not differ significantly between cohorts." In consideration of significance- Even if it did not differ significantly, did time to disposition differ at all? Kindly state the difference if any (means)and p-value. Perhaps some issues are not statistically significant but might be clinically significant.**

We would like to thank the reviewers for their comment. For additional clarity, we have added the medians with a p-value for clarification (Results, line 203-204).

8. **Authors should confirm- Is there the possibility that point of care focused assessment sonography for trauma might have been missed in the registry records? Bedside/ED ultrasound scan in some LMICs, by experience, is usually not ordered or documented as a radiological "procedure" or standard assessment.**

We would like to thank the reviewers for their question. Point-of-care ultrasound was not available at any of the study sites during the study period. For increased clarity, we have added this information to the discussion (Discussion, line 231-232).

9. **Lines 211-212 "In Cameroon, our data suggest that both under-prescription and barriers to imaging completion play a role in the limited utilization of radiology." Underprescription does not appear to be the focus of this study, and should not be emphasized.**

We would like to thank the reviewers for their comment. While we fully agree that assessment of imaging underprescription is not the primary objective of our study, we do believe that our data are highly suggestive that underprescription is occurring in this population. We felt that these ancillary findings are important to note briefly as this may also substantially contribute to poor outcomes and should be taken into account when considering development of future prospective clinical trials.

10. **The Limitations are excellent observations. Generalizability to other healthcare systems *will* be imperfect, and should be acknowledged as such**

We would like to thank the reviewers for their comment. We agree and have modified our language to reflect this limitation (Discussion, line 307).

11. **Authors should consider including a reference to the Lancet Commission on Diagnostics as there is much alignment with the intent of this paper- and for suggestions of set standards**

We would like to thank the reviewers for their insightful suggestion. We have added this reference to our manuscript (Discussion, lines 256-258, reference 32).

12. **Please edit reference 10 (Line 296)**

Thank you. We have modified reference 10.

13. **Figure 1- showing what was not completed in Figure 1 would help clarify what specifically is NC (to mirror the completed. Kindly add this in for more clarity on the part of the reader.**

We would like to thank the reviewers for their comment. We have modified Figure 1 accordingly and have provided additional detail discussing the differing distribution of performed and non-performed imaging (Results, lines 161-163).

14. **Authors should also kindly briefly comment of overutilization of imaging with financial and environmental implications that typically can occur in high resource settings, and where the balance should be, in the discussion.**

We would like to thank the reviewers for this insightful suggestion. We have added a brief discussion on maintaining this balance to the discussion section of our manuscript (Lines 277-282).

15. **Finally, the authorship order raises a few concerns of LMIC authors being "stuck in the middle." Could authors consider co-first authorship based on other contribution considerations, co-senior authorship, or at the minimum, having a Cameroonian corresponding author, as this work is done on data from Cameroon? there is some reflexivity required here, and the authorship team would do well to consider carefully the inclusive placement of LMIC authors in the hierarchy of authorship.**

We would like to thank the reviewers for their comment. We agree this is a critical issue. If permissible by the manuscript, Drs. Christie and Chichom should be listed as co-senior authors.

Reviewer 2:

1. **While in the text of the manuscript, it was mentioned correctly that findings are associations, the title of the manuscript mistakenly shows a causality: "Failure to Receive Prescribed Imaging Portends Increased Early Mortality after Injury in Cameroon", "increased" should be replaced with "associated with increased"**

We would like to thank the reviewers for their insightful comment. We agree and have modified the title to avoid implying causality. It now reads "Failure to Receive Prescribed Imaging is Associated with Increased Early Mortality after Injury in Cameroon."

2. **Methods: "Verbal consent was obtained by research assistants." It is not clear how verbal consent was obtained as participants were injured and usually it is not possible to take consent from people who suffer from a recent injury, also if for a participant the outcome of the injury was death, how was the consent obtained?**

We would like to thank the reviewers for their comment. We have added additional detail to the manuscript as described in detail in our response to Reviewer 1, comment 2.

3. **Methods: In this study, data was collected on paper registry forms which were then entered into a cloud-based server. The information about data collection was limited but as I understood this form was a usual history-taking form designed for trauma patients and the data was collected from patients as a routine for the personnel, while they were admitted and medical procedures were on-process. So, the quality of the data is questionable. Especially, it may be biased for underreporting from severely injured patients. For example, about the results of table 1: maybe severely injured patients did not complete the imaging because maybe their hemodynamic status was unstable and they were unable to perform imaging and so due to their instability maybe they did not answer having access to LPG or having a cell phone or having a past medical history. Authors should clarify how they addressed these possibilities of bias.**

We would like to thank the reviewers for their comment. Analogous to trauma registry data methodology in developed settings, information regarding patient demographics was collected by research from both patients and surrogates throughout the hospital stay. Although use of surrogates and updated data collection after the acute phase of assessment and evaluation decreases data missingness, we fully acknowledge that severely injured patients without families may have increased missingness of certain demographic variables. For clarity we have emphasized this in the limitations section (Limitations, lines 295-297). However, we do not expect this to skew the results as "unknown" is also an option for all variables mentioned. To address potential bias, associations between imaging completion and death were adjusted for injury severity.

4. **Methods: For adjusting logistic regression, it should be stated how the injury severity was assessed. Also, who did assess the severity?**

We would like to thank the reviewers for their comment. As in many LMICs, routine calculation of conventional anatomic injury scores such as ISS is not feasible in Cameroon due to limited availability of cross-sectional imaging. As part of the CTR, the clinical care team estimates an Abbreviated Injury Scale (AIS) for each anatomic region based on physical exam findings. The Highest Estimated Abbreviated Injury Scale (HEAIS) is used as the primary metric of injury severity in our cohort. Clinical assessment of injury severity using HEAIS strongly predicts hospital mortality and far exceeds conventional scoring systems (Revised Trauma Score, Kampala Trauma Score) in feasibility [Yost MT, Carvalho MM, Mbuh L, Dissak-Delon FN, Oke R, Guidam D, Nlong RM, Zikirou MM, Mekolo D, Banaken LH, Juillard C, Chichom-Mefire A, Christie SA. Back to the basics: Clinical assessment yields robust mortality prediction and increased feasibility in low resource settings. PLOS Glob Public Health. 2023 Mar 29;3(3):e0001761. doi: 10.1371/journal.pgph.0001761. PMID: 36989211; PMCID: PMC10057736.]. We have included a brief discussion of highest estimated abbreviated injury severity score, the scale used in our paper (Methods, lines 125-128).

5. **Please add strengths of the study prior to its limitations.**

We would like to thank the reviewers for their comment. As suggested, we have outlined several of the key strengths of the study prior to discussing the limitations (Discussion, lines 287-292).

6. **Insurance has a great impact on healthcare utilization. In this study, insurance coverage status was not investigated and also was not mentioned in the discussions.**

We would like to thank the reviewers for their comment. We have added information on Cameroon's current healthcare economics structure, including its fee-for-service model and minimal availability of health insurance (Introduction, lines 67-69).

7. **The manuscript did not mention the references for physicians defining the indications of radiologic imaging requests that were authorized or published by Cameroon's ministry of health or other related health sectors. If there are no local guidelines, the usual references for physicians should be mentioned briefly.**

We would like to thank the reviewers for their comment. There are currently no uniform practice guidelines routinely followed in Cameroon regarding indications for imaging in trauma patients. Developing contextually appropriate imaging guidelines may provide a target for streamlining evidenced based care and improving outcomes after imaging in Cameroon. For clarity, we have added this additional information to the manuscript (Discussion, lines 282-286).

8. **In the background section was mentioned: "As part of a larger quality improvement initiative and to assist priority setting for policymakers, we evaluated trauma outcomes among patients who did and did not receive indicated imaging in the Emergency Department (ED). We hypothesize that receiving imaging is associated with increased early injury survival." While these two sentences provided valuable information, I recommend presenting exactly and clearly the aim of the study, instead.**

We would like to thank the reviewers for their comment. We have clarified our study's aims in Lines 84-86.

9. **Introduction: "Over 80% of Cameroonian imaging centers are limited to radiographs and/or ultrasound." This sentence needs a reference.**

We would like to thank the reviewers for their comment. We have added the citation to the manuscript (Reference section, item 15, line 76).
