# Supplementary material for: Failure to receive prescribed imaging is associated with increased early mortality after injury in Cameroon
Source: PLOS Glob Public Health. 2023 Aug 18;3(8):e0001951. doi: 10.1371/journal.pgph.0001951 (PMC10437996; doi:10.1371/journal.pgph.0001951)
Supplement: Attachment — Submitted filename: Response to Reviewers.pdf [file pgph.0001951.s003.pdf]

UNIVERSITY OF CALIFORNIA, LOS ANGELES **UCLA**

BERKELEY • DAVIS • IRVINE • LOS ANGELES • MERCED • RIVERSIDE • SAN DIEGO • SAN FRANCISCO SANTA BARBARA • SANTA CRUZ

DEPARTMENT OF SURGERY
DAVID GEFFEN SCHOOL OF MEDICINE, UCLA
10833 LE CONTE AVE 72-215 CHS
LOS ANGELES, CALIFORNIA 90095-6904
PHONE: (310) 267-9607
FAX: (310) 267-3590

July 20, 2023

Dear Dr. Alayande,

Thank you for your review of our manuscript "Failure to Receive Prescribed Imaging is Associated with Increased Early Mortality after Injury in Cameroon" for PLOS Global Public Health. We appreciate the kind feedback from the reviewers following our first set of revisions, as well as the commitment to an equitable authorship distribution. We have added the clarification in our manuscript's acknowledgement selection regarding the co-authorship between Drs. Chichom-Mefire and Christie, also outlined below.
We appreciate your time and would like to express our thanks in your ongoing consideration of our manuscript for publication in PLOS Global Public Health. Please do not hesitate to contact us for any further questions or comments.

Sincerely,

Matthew Driban, BS (Corresponding Author)

Please see below for an itemized response to each of the reviewers' comments.

1. **In response to your desire that Alain Chichom-Mefire and S. Ariane Christie be joint senior authors, kindly add a note in your acknowledgements to this effect- "Alain Chichom-Mefire and S. Ariane Christie are joint senior authors." This is the only change needed to move this forward.**

Thank you for ensuring the delineation of senior authorship is clear. We have added the requested note to our acknowledgements (Line 311).

2. **In view of the singular concern by Reviewer #2, I can confirm that the location of the supplementary file named "CTR_Radiology_PLOS_Deidentified.xls" mentioned in your response is at the end of the revised document following the figures and just before the manuscript with tracked changes. Thank you for including this. As the reviewer noted, this is particularly important to ensure the transparency and reproducibility of your work.**

Thank you for this clarification and the commitment to transparency and reproducibility.
